# Physical evidence of direct antagonistic interactions between trematodes in the host gut: the kiss of death?

**DOI:** 10.1007/s00436-023-07883-7

**Published:** 2023-05-22

**Authors:** Bronwen Presswell, Jerusha Bennett, Xuhong Chai, Robert Poulin

**Affiliations:** Department of Zoology, University of Otago, PO Box 56, Dunedin, 9054 New Zealand

**Keywords:** Competition, *Dinosoma synaphobranchi*, *Glomericirrus macrouri*, Hemiuridae, Macrouridae

## Abstract

Although within-host competition among parasites if often assumed to occur based on statistical patterns, actual physical evidence of direct antagonistic interactions between parasites, either intraspecific or interspecific, is very rare. Here, we report such evidence between and within two species of hemiurid trematodes infecting the deep-sea grenadier fish *Coryphaenoides subserrulatus*. We found pairs of worms attached together, with one worm using its ventral sucker against another worm, and sucking out a large protuberance on the victim. We also found single worms showing clear signs of past attacks. There was no evidence that these interactions were more common at high intensities of infection, where the conditions would be expected to be more conducive to competitive interactions. Our findings provide evidence that trematodes may cause some harm to co-occurring individuals, suggesting a direct form of interference competition among intestinal helminths.

**Supplementary Information:**

The online version contains supplementary material available at 10.1007/s00436-023-07883-7.

## Introduction

In the limited volume of a vertebrate gut, helminth parasites are assumed to compete for space and nutrients. Evidence of interspecific competition often relies on statistical patterns; for instance, a negative correlation between the abundance of two helminth species across different host individuals can suggest that high abundance of one species prevents another from also achieving high abundance (Dobson 1985; Poulin 2001; Behnke et al. 2005). Competition among conspecific individuals, i.e., intraspecific competition, is also usually demonstrated using infection data. For example, the proportion of individuals successfully establishing in a host during experimental exposure can decline with increasing infection dose, suggesting that competition among individuals escalates at high intensity of infection and results in more individuals failing to establish (Brown 1986; Ashworth and Kennedy 1999).

All these approaches, however, provide only *indirect* evidence of competition. The patterns observed may have other explanations. For example, a greater number of conspecific parasites in one host can trigger a stronger immune reaction, leading to a higher proportion of individual parasites being expelled (Cox 2001). This would be a result of host-mediated processes, and not actual competition between the worms. In contrast, *direct* evidence of competition between parasites, involving empirical demonstration of physical interactions between individual worms with negative consequences for at least one of them, is generally lacking. As an exception, interspecific antagonistic interactions between species of larval trematodes sharing the same snail intermediate hosts have been well documented, with rediae of some species known to attack and kill individuals (usually sporocysts) of other species (Sousa 1993; Leung and Poulin 2011). However, similar competitive interactions, either between or within species, among adult trematodes in their definitive hosts are rarely observed.

Here, we provide evidence of antagonistic interactions, consistent with a form of active competition, between individual trematodes parasitic in the gastrointestinal tract of fish hosts (*Coryphaenoides subserrulatus*, family Macrouridae). The interactions we observed suggest potential physical damage inflicted by one worm on another. To our knowledge, such observations are rare (see examples in ‘Results and discussion’), and the present findings provide some support in favour of the existence of competitive interactions that may be detrimental to the recipient and that could play a small role in regulating the size of parasite infrapopulations.

## Material and methods

Material used here comes from samples obtained for an earlier study (Chai et al. 2022) that were collected during a January-February 2020 National Institute of Water and Atmospheric Research (NIWA) cruise along the Chatham Rise, a submarine ridge east of New Zealand’s South Island. All seven individuals of the fish species *Coryphaenoides subserrulatus* were trawled from the same location (44°30′S, 178°30′W), at a depth of 975–1000 m. They were dissected right after capture, and helminths were recovered from the gastrointestinal tract and immediately preserved in 70% ethanol until their return to the laboratory.

Trematodes were identified morphologically, as much as possible. Representative specimens were stained with acetic acid iron carmine, dehydrated in an ethanol series, cleared in clove oil, and permanently mounted with Canada balsam for light microscopy. They were identified to the lowest taxonomic level possible using various keys (see Chai et al. 2022 for details). Two species of hemiurids, *Dinosoma synaphobranchi* and *Glomericirrus macrouri*, were identified, with the former being more abundant than the latter (based on molecular data, see below). Since the two species are quite similar in morphology and not all individuals were stained and measured or sequenced (see below), hereafter we pool their data and report prevalence and intensity values for hemiurids and not for each species separately. Some hemiurids (of the same or different species) were either attached to each other in pairs, in what looked like one individual ‘attacking’ another one, or they were unattached but showed signs of past attacks. For simplicity, hereafter, we refer to these individuals as either attackers or victims.

To confirm species identification, molecular tools were also used on two individuals, in this case an attacker and a victim from the same attached pair, as well as 29 individual hemiurids that were not attached together and showed no sign of past attack. Following DNA extraction, a fragment (approximately 600 bp length) of the 28S gene was targeted using the primers T16 and T30 (Harper and Saunders 2001). PCR conditions are described in Presswell and Bennett (2020). Sanger sequencing was performed by the Genetic Analysis Service, Department of Anatomy, University of Otago (New Zealand). Sequences were imported into Geneious Prime 2022.0.2 (https://www.geneious.com), trimmed using default settings, and manually edited for ambiguous base calls. They were uploaded to the BLAST online search tool (https://www.ncbi.nlm.nih.gov) and compared to publicly available sequences on GenBank to confirm species identification; see Chai et al. (2022) for details.

Note that in addition to the two hemiurid species, the fish were also infected with an unidentified adult Lecithasterinae trematode (one fish infected with 2 worms), an adult bothriocephalidean cestode (one fish infected with 2 worms), and juvenile *Anisakis* spp. nematodes (three fish infected with 1 worm each). These parasites are not further considered here.

To test for possible intensity-dependence, we computed the Spearman’s correlation coefficient between the percentage of worms that were victim of attacks (combining those fixed while being attacked and those showing signs of past attack) and the number of hemiurid worms per individual host.

## Results and discussion

**Fig. 1:**
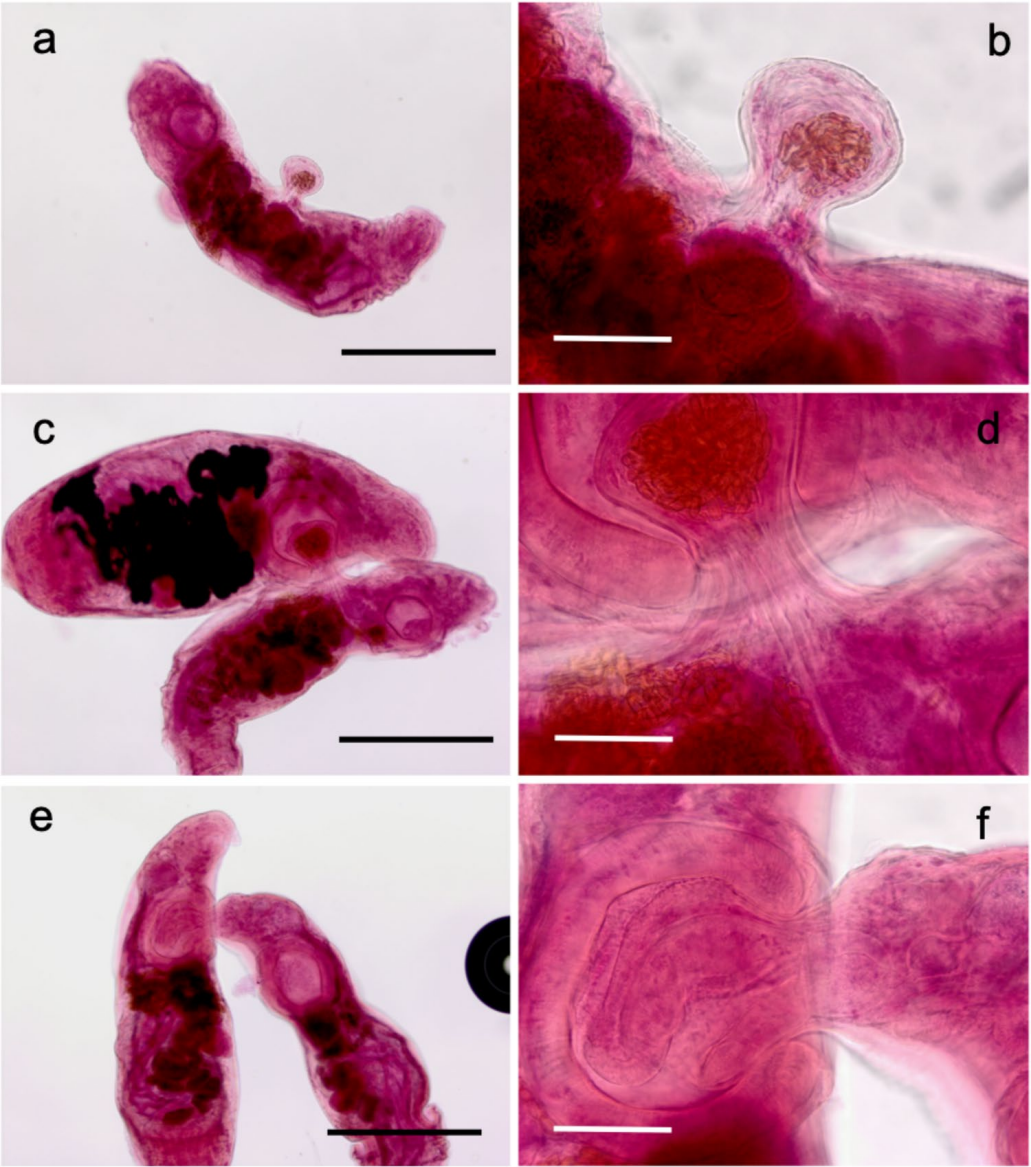
Examples of hemiurid trematodes attacking other worms and impact of such attacks. **a** Single worm showing a lateral bleb; another, lateroventral one is seen out of focus on the left. **b** Close up of bleb showing part of uterus sucked in with tissue, but not apparently damaged. **c** Pair of worms, one attached by dorsal surface to another’s ventral sucker. **d** Close up of same showing uterus sucked into the other’s ventral sucker, again without apparent damage. **e** Pair of worms with anterior end of one sucked into ventral sucker of the other. **f** Close up showing oral sucker inside ventral sucker of the other worm. Scale bars: **a**, **c**, **e**, = 500 μm; **b**, **d**, **f** = 100 μm

Of the 7 individual fish *C. subserrulatus* examined, 6 were infected with between 1 and 84 hemiurids (Table 1). In several cases, two individual worms were attached, with the attacker latching on the victim with its ventral sucker and exerting enough suction to produce a bleb, i.e., a large, rounded, blister-like protuberance on the body of the victim (Fig. 1). Although we did not observe lethal damage, such as a breach of the tegument, tissues from the victim including the uterus were often sucked into the bleb (Fig. 1). It is not unlikely that damage is caused to internal tissues as they pass through the narrow neck of the bleb (Fig. 1b, d). We also observed single worms, no longer attached to an attacker but showing signs of past attacks, i.e., one or more blebs on their bodies (Fig. 1).

**Table 1:** Number of worms (Hemiuridae) attached in pairs, number of single worms showing signs of past attacks, and number of single worms with no sign of past attack, in each of six individual hosts (*Coryphaenoides subserrulatus*, Macrouridae), listed in order of increasing intensity of infection

Host ID	No. pairs of attached worms	No. single worms with signs of past attacks	No. single worms with no sign of past attack	Total no. worms
CSU4	0	0	1	1
CSU8	2	0	11	15
CSU5	3	0	16	22
CSU3	2*	1	20	26
CSU7	1	4	43	49
CSU6	6	1	71	84

*One group was actually a triad, with one worm attacking a second worm, which was itself attacking a third worm

The pair of individuals sequenced revealed that the attacker was *Dinosoma synaphobranchi*, while the victim was *Glomericirrus macrouri*. Interestingly, whereas *G. macrouri* is a common parasite of macrourid fishes like our study species (Gibson and Bray 1986), *D. synaphobranchi* had never been recorded in macrourids prior to our study (see Klimpel et al. 2009; Chai et al. 2022), and may not have the same coevolutionary history with macrourids as *G. macrouri*. However, since only worms from one pair of attacker and victim were sequenced, the exact identity of worms in other pairs is unknown, and therefore, both intraspecific and interspecific interactions may occur.

**Fig. 2:**
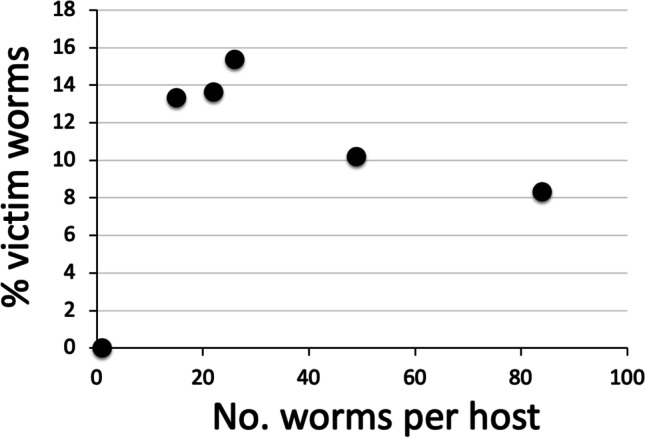
Percentage of individual worms that were victim of attacks by another worm (either attacked at the time of fixation or showing signs of past attacks) as a function of the number of worms per individual host, for hemiurid trematodes infecting the grenadier fish *Coryphaenoides subserrulatus*

A priori, one might expect these sorts of antagonistic interactions to be more frequent at high intensities of infection, where competition for space in the host gut should be more intense. However, we found no evidence of intensity-dependence in the frequency of attacks (Fig. 2), as the correlation between the percentage of worms victim of attacks and the number of hemiurid worms per individual host was negative but not significant (excluding the fish with a single hemiurid: *r*_s_ = −0.60, *N* = 5, *P* = 0.285). Although a negative relationship with intensity of infection would be counterintuitive if it were significant, there are too few infected fish in our sample for any conclusive evidence.

There are at least two reasons why the low number of worms we observed either in the process of being attacked or showing signs of past attacks might be underestimating the true frequency of physical attacks. First, the protuberance left behind by an attack might be short-lived, even if the internal damage caused may be severe, leaving no visible signs that we could detect. Second, worms that were victims of attacks may be more likely to die and/or be dislodged, and thus pass out of the host’s gut, leaving no trace of their presence.

We acknowledge that it is possible that the attachment of one worm onto another might not have occurred in the fish intestine, but instead be an artefact of the crowded conditions in the dish where the worms were placed post-dissection for a minute or two, prior to being fixed in ethanol. However, we find this unlikely since the worms spent little time in the dish before being killed, probably too little time to attach to another worm, exert prolong suction, and detach leaving a large protuberance on the victim. Furthermore, similar observations have been made before, also involving one trematode attaching to another with its ventral sucker (Fried and Lang 1971; Jacobson et al. 1974). We have also observed a similar phenomenon among other hemiurids, *Parahemiurus* sp., in the fish *Seriolella brama* caught off the New Zealand coast, with a large portion of one worm sucked into the ventral sucker of another (J. Bennett, personal observation; see video in Supplementary Material). This was observed during dissection and not after, the attack having definitely taken place within the host gut. Overall, we are confident that these antagonistic interactions truly occur within the host.

Competition between members of the same species can take two forms. First, exploitative competition occurs passively when one individual, simply by consuming resources, reduces the resource pool available for others. Secondly, interference competition involves an individual actively preventing others from accessing resources, usually through some form of aggressive or antagonistic interaction (Townsend et al. 2008; Gorter et al. 2021). The phenomenon we document here aligns more closely with the latter form of competition. Interference competition among parasitic helminths has been suggested earlier, with chemical factors secreted by cestodes believed to retard the growth of conspecifics sharing the same host (e.g., Cook and Roberts 1991). However, along with the earlier examples discussed above (Fried and Lang 1971; Jacobson et al. 1974), our findings represent more direct physical aggression by trematodes possibly causing harm to their conspecifics. Whether the attacks we observed actually cause the victim to die, or to detach from the intestinal wall and pass out of the host, remains to be demonstrated. Nevertheless, the size of the blebs resulting from these interactions suggests that the victim incurs some form of harm that may reduce competition for space or other resources among remaining worms, and thus benefit the attacker.

## Supplementary information

ESM 1 MOV 65.1 MB

## Data Availability

All data used in this study are shown in Table 1.
